# The Classification of Alcoholics: Typology Theories From the 19th Century to the Present

**Published:** 1996

**Authors:** Thomas F. Babor

**Affiliations:** Thomas F. Babor, Ph.D., M.P.H., is a professor and scientific director at the Alcohol Research Center, Department of Psychiatry, University of Connecticut School of Medicine, Farmington, Connecticut

**Keywords:** AOD dependence, disorder classification, Jellinek typology, historical review, AOD use pattern, personality trait, AODR (alcohol and other drug related) disorder, comorbidity, etiology, AOD associated consequences, treatment research

## Abstract

Alcoholics differ in many of their personal and drinking-related characteristics, and for the past 150 years, clinicians and researchers have tried to categorize alcoholics based on these differences. Such typologies can advance our understanding of alcoholism as well as improve treatment of the disease. The history of alcoholism typology can be divided into three periods: the prescientific period, the Jellinek era, and the post-Jellinek era. During the prescientific period, physicians—especially those specializing in treating mental illnesses and addictions—developed numerous typologies, building primarily on clinical observation, anecdotal evidence, and armchair intuition. E.M. Jellinek has been credited with creating the first scientific typology that was developed into a comprehensive theory of alcoholism as a disease. The typologies that have evolved since Jellinek’s landmark work have been derived mainly from empirical research data. Despite the wide variety of methodological approaches used, it appears that subtypes from all typologies developed since the 19th century can be classified into two major categories, the Apollonian and Dionysian subtypes.

Although alcoholism often is treated as a unitary disorder that can be described by a single disease label, ample evidence indicates that alcoholics differ in a wide variety of defining characteristics, such as drinking patterns, type of dependence, genetic predisposition, personality traits, and antecedent psychiatric disorders. Recognition of this heterogeneity has led to attempts to develop alcoholism typologies—that is, to classify groups of alcoholics according to defining characteristics—in order to better understand the etiology of alcoholism (i.e., the mechanisms leading to the disease), improve treatment, and advance the theoretical framework for alcoholism and its consequences.

This article traces the clinical and scientific thinking about alcoholism typologies during the past 150 years. During this time, the history of typological thinking about alcoholics can be roughly divided into three periods: the prescientific period of clinical speculation (1850–1940), the Jellinek era of review and synthesis (1941–1960), and the post-Jellinek period of increasingly sophisticated empirical research (1960–present). To the extent that ideas do not develop in a vacuum, this history provides an interesting example of how “invisible colleges” of like-minded thinkers are capable of advancing knowledge both as groups and individuals. The article also demonstrates that despite the plethora of alcoholism typologies developed over time and the variations among them, recurring traits in the drinkers’ personalities appear to exist among the typologies, thereby allowing alcoholism subtypes to be separated into two major categories, the Apollonian and Dionysian subtypes.

## Why Is Alcoholism Typology Important?

The urge to classify objects, ideas, and people into meaningful categories, or types, is a basic characteristic of human nature. When types are organized into a system according to definitional rules and practical applications, the classification is called a typology. Although the tendency to classify people undoubtedly serves an important medical function (e.g., knowing that a particular patient with liver disease is an alcoholic may help clinicians more effectively manage that patient’s disorder), the common propensity to reduce individual differences among people to simplistic stereotypes also can distort our perceptions of social reality. Alcoholics commonly have been associated with dysfunctional stereotypes, as evidenced by the variety of pejorative terms used in different languages to describe them. For example, vagabonds and homeless people with alcohol-related problems have been referred to as “Bowery bums” and “Skid Row alcoholics.” Other historic, derogatory terms have included “sot,” “wino,” “rummy,” and “lush.” However, when looking beyond this oversimplification of popular culture to the history of medicine and psychiatry, it is clear that typologies based on the organization of clinical information through diagnostic classification, medical nomenclature, and clinical subtyping have advanced our theoretical knowledge as well as the art of healing.

## The Prescientific Period (1850–1940)

It would seem logical to begin a discussion of the history of typology with E.M. Jellinek’s classic work on the different “species” of alcoholism (1960*a*, *b*), which is widely considered to be the first scientific alcoholism typology. Various historians, such as Mark Lender (1979), however, have pointed out that many 19th and 20th century “alienists” (i.e., physicians specializing in treating mental illnesses and addictive disorders) had a remarkably sophisticated appreciation of alcoholism. Moreover, by studying the evolution of alcoholism typologies, current researchers can place Jellinek’s ideas and subsequent thinking into a broader perspective.

The prescientific period of alcoholism typologies roughly extends from William Carpenter’s description in 1850 of different types of “oinomania,” or wine mania (Carpenter 1850), to the psychoanalytic and character-based theories of the 1930’s. In many countries, alcoholism emerged as a major public health problem during the 19th century, just when medicine and psychiatry were developing as modern professional guilds. Thus, it is no coincidence that some of the leading physicians in countries such as France, England, Germany, and the United States devoted considerable attention to studying alcoholism. According to a review of the world alcohol literature, 39 classifications of alcoholics were developed between 1850 and 1941 (Babor and Lauerman 1986). Most of these typologies were published by alienists in books and scholarly journals.

One of the earliest and most influential classifications was introduced in Carpenter’s 1850 essay entitled *On the Use and Abuse of Alcoholic Liquors in Health and Disease*. Quoting extensively from the *Report of the Glasgow Lunatic Asylum* published in 1842, Carpenter proposed three categories of oinomania: acute, periodic, and chronic. In the acute form, the desire to drink occurs suddenly, but the disease rarely progresses beyond irregular intoxication. The periodic form is characterized by a pattern of binge drinking that becomes progressively more severe and damaging. In the chronic form, the desire for alcoholic stimulation becomes an overwhelming preoccupation that precipitates constant alcohol consumption.

**Figure:**
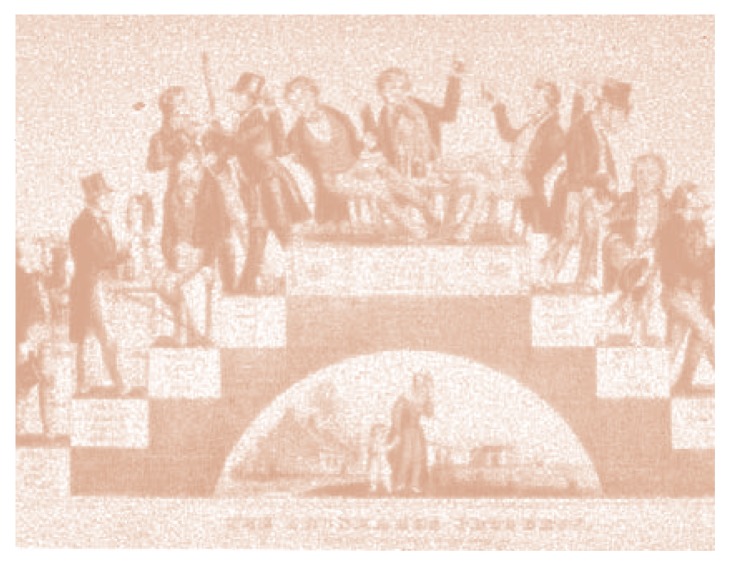
The unitary disease concept, as illustrated in “The Drunkard’s Progress,” by Nathaniel Currier. Typology theorists believe this is an inadequate representation of the heterogeneity of etiologies and drinking patterns. Reproduced with permission from the *Journal of Studies on Alcohol*. © Alcohol Research Documentation, Inc., Rutgers University Center of Alcohol Studies.

Twenty years after Carpenter’s essay, several American physicians specializing in the care of alcoholics organized the Association for the Study of Inebriety, which promoted the disease concept of alcoholism, advocated the establishment of specialized hospitals for inebriates, and supported the scientific study of inebriety^1^ (Lender 1979). In 1876 the association established the *Quarterly Journal of Inebriety*, which, over a period of 38 years, published numerous articles by leading physicians from the United States and abroad about the different forms of alcoholism. Similar societies formed in England, under Norman Kerr’s leadership, and in France, under Valentin-Jacques Magnan’s direction. Through national and international meetings and a wide circulation of books and journals, the writings of these physicians and alienists became instrumental in defining the medical response to what was considered the disease of inebriety.

In 1893 Kerr, who also was an honorary member of the American association, published the influential textbook *Inebriety and Narcomania*, which divided inebriates into two groups, periodic and habitual (Kerr 1893). Periodic inebriety is characterized by intense drinking or craving for alcohol interspersed with periods of abstinence. For some alcoholics, the drinking periods are determined by internal cues, such as the onset of menses in women. For others, external opportunities, such as a worker’s payday or sailor’s shore leave, govern the periodicity of inebriety. Intervals of intense nervous irritability and depression commonly precede the drinking periods. Periodic inebriety often takes the form of temporary insanity, in which the drinker’s behavior is characterized by mania, violence, or impulsive criminal behavior.

Habitual inebriety begins as a “voluntary indulgence” that eventually crosses the line between the physiological and the pathological, resulting in a deterioration of physical and mental abilities. Both habitual and periodic inebriety may manifest themselves in different ways, leading to a further classification of inebriates as social and unsocial. Social inebriates drink openly with other drinkers, whereas unsocial, or solitary, inebriates shun the company of others and tend to drink secretly, often because of “neurasthenia” (i.e., exhaustion of the nervous system).

In 1911 Thomas Crothers, cofounder of the Association for the Study of Inebriety, presented another alcoholism typology in his book *Inebriety: A Clinical Treatise on the Etiology, Symptomology, Neurosis, Psychosis and Treatment.* His classification, based on years of clinical experience, resembled Kerr’s classification but included three types: the continuous drinker, the explosive inebriate, and the periodic drinker (Crothers 1911). The first type, the continuous drinker, is characterized by a lengthy preliminary period of moderate drinking leading to the “gradual growth and cultivation of the drinking impulse” (p. 40). The explosive inebriate uses alcohol infrequently, usually in response to some precipitating cause, and becomes extremely intoxicated. The third type, the periodic drinker, includes several subcategories, such as dipsomaniacs, who are “marked by an insane overpowering impulse which is a veritable mania” (p. 41). In addition, the periodic drinker is influenced by environmental factors, mental stress, and physical conditions. The abrupt onset and cessation of drinking resemble epilepsy and other convulsive disorders, “with distinct physical causes and conditions not under the control of the will” (p. 71). Crothers proposed that emotional factors can be both the cause and consequence of periodic drinking, with intense excitement or depression frequently preceding intoxication and “melancholia” and suicidal impulses often following it.

In discussing the general causes and conditions favoring inebriety, Crothers (1911) also classified alcoholism as either acquired or hereditary. People with acquired inebriety often have histories of physical disorders, particularly dyspepsia (i.e., indigestion), bad nutrition, and exhaustion from unhygienic living conditions or stressful work environments. Conversely, hereditary causes include constitutional conditions, such as distinct neurotic and psychopathic disorders that often are traceable to ancestors.

As in the United States and England, prominent French alienists during the late19th century also began writing about the increasing numbers of alcoholics found in the nation’s insane asylums. In his book, *Hérédité et Alcoolisme*, Paul Maurice LeGrain (1889) incorporated the major psychiatric theories of the day into a comprehensive alcoholism typology that included three types of alcoholics: morally insane alcoholics, weak-willed alcoholics, and dipsomaniacs. The first type, morally insane alcoholics, have a poorly developed moral sense and thus do everything in excess. Their frequent intoxication often results in accidents and violence. The second type, weak-willed alcoholics, have an adequate moral sense but lack will-power. These alcoholics drink either because they like the taste of alcohol (“par gout”) or by habit (“par entrainement”). The third type, dipsomaniacs, are impulsive drinkers whose will-power dissolves in alcohol. In addition, all three types suffer from defective mental states brought on primarily by inherited mental degeneracy, which was thought to be cumulative in certain families.

A decade later, physicians Henri Triboulet and Félix Mathieu (1900) distinguished between dipsomaniacs, hereditary regular alcoholics, and non-hereditary habitual drinkers, suggesting that the latter respond well to simple advice at an early stage and to voluntary commitment to a special asylum at a later stage.

A common theme in the French medical literature of the time was the description of a less socially disruptive form of alcoholism epitomized by Dromard’s (1902) term “les alcoolisés non-alcooliques” (chronically alcoholized nonalcoholics). These regular drinkers develop progressive habituation to alcohol’s toxic effects, followed by an irresistible need to drink. Morning drinking relieves mild withdrawal symptoms, and the person’s life becomes centered around the use of small doses of alcohol throughout the day. Eventually alcohol’s cumulative effects lead to major medical complications and organic brain disorders.

In one of the first books devoted exclusively to alcoholic subtypes, English physician Hugh Wingfield explored the nature, causes, and treatment of alcoholic subtypes in his 1919 treatise on *The Forms of Alcoholism and Their Treatment*. Like his predecessors, Wingfield collected much of the information from his own experiences with patients. He proposed four main varieties of alcoholics: pseudodipsomaniacs, chronic sober alcoholics, chronic inebriate alcoholics, and true dipsomaniacs (Wingfield 1919). Pseudodipsomaniacs drink in great excess, usually in bouts lasting a week or more, as a result of craving induced by an initial drink of alcohol. Chronic sober alcoholics are characterized by daily drinking over relatively long periods of time. They are infrequently intoxicated and crave alcohol only if it is partially or completely withheld. Chronic inebriate alcoholics drink regularly and are almost always intoxicated. Finally, true dipsomaniacs experience spontaneous craving and engage in short drinking bouts.

In questioning the value of “compulsory restraint in a retreat for long periods,” Wingfield (1919, p. 42) proposed specific treatments for different types of alcoholics. For pseudodipsomaniacs and true dipsomaniacs, he recommended administering small doses of apomorphine to provide temporary relief of craving and morphia to treat intense depression. Chronic alcoholics should first be given diminishing doses of alcohol to reduce the risk of delirium tremens before being treated with drugs and “suggestion.” The drug of choice was atropine, given in conjunction with strychnine. Suggestion, especially under light hypnosis, was designed to “lessen the risk of relapse long after treatment is ended” (p. 68). According to Wingfield, suggestion reduces “haunting ideas of drink,” increases the patient’s will power, and sometimes brings to light repressed memories, “effecting a real cure thereby” (pp. 69–70).

Wingfield’s reference to repressed memories indicates the growing influence of psychoanalytic theory, which argued that alcoholism was merely the symptom of an underlying neurosis. Psychoanalyst Robert Knight (1938) developed these ideas further, proposing three types of alcoholics: essential alcoholics, reactive alcoholics, and symptomatic drinkers. The first type, essential alcoholics, are characterized as psychopaths with an oral fixation and a conflict between feminine passivity and masculine strivings. They experience an early onset of alcohol problems and do not perform well in school or at work. In contrast, reactive alcoholics usually begin drinking in response to a precipitating event and respond better to treatment, in part because they are better adjusted initially. The third type, symptomatic drinkers, experience prominent neurotic or psychotic symptoms that are responsible for their drinking.

In contrast, typology theory in Germany was influenced not so much by psychoanalytic ideas as by constitutional theories that explained drinking behavior based on physique and temperament. Ernest Kretschmer (1924), for example, proposed two groups of chronic alcoholics: a cyclothymic type whose drinking results from a pliable, gregarious disposition, and a schizoid type, who uses alcohol to relieve internal stress. With the further development of constitutional theories during the 1930’s, typological formulations were used to justify the involuntary surgical sterilization and removal of “hereditary” alcoholics to concentration camps during the height of the Nazi era (Babor and Lauerman 1986).

### The Significance of Early Typologies

As this brief review demonstrates, the early typologies were unsystematic, based primarily on clinical observation and anecdotal evidence, and lacked an empirical foundation, thus leading to a confusing array of concepts and nomenclature. Moreover, they did not lead to the development of theories explaining the etiology, manifestations, and consequences of alcoholism, because they did not propose verification procedures to test assumptions and predict behavior.

Despite these shortcomings, the early attempts to differentiate and classify alcoholics had a positive influence on the development of alcohol studies. For example, they led to the identification of important defining characteristics of alcoholic subtypes, such as family history, psychopathology, drinking patterns, personality factors, and physical consequences. These early typologies also introduced the concept of treatment matching;^2^ inspired some crude attempts at empirical investigation; and suggested that the etiology, symptomatology, and natural history of alcoholism were complex phenomena. Finally, they set the stage for the development of more sophisticated theories, such as those developed by Jellinek.

## The Jellinek Era

In 1941 psychiatrist Karl Bowman and biometrist E.M. Jellinek wrote a comprehensive review of the alcoholism treatment literature for the newly established *Quarterly Journal of Studies on Alcohol* (Bowman and Jellinek 1941). Their review, “Alcohol Addiction and Its Treatment,” contained a masterful integration of 24 typological formulations that had appeared in the world alcohol literature prior to 1940 and which formed the basis for the most detailed alcoholism typology to date. Using a hierarchical classification approach modeled after the way botanists identify genera and species, this typology began with two broad categories defined by the pattern of drinking (i.e., steady and intermittent, which was further differentiated into periodic and irregular). These groups were further subdivided according to the etiology of the disease into subtypes resulting from internal (i.e., endogenous) or external (i.e., exogenous) causes, resulting in four major categories—primary alcoholics, steady endogenous symptomatic drinkers, intermittent endogenous symptomatic drinkers, and stammtisch drinkers—and several minor categories that encompassed the subtypes identified by previous theorists. The four major subtypes were described as follows:

- Primary or “true” alcoholics are characterized by their immediate liking for alcohol’s effects, the rapid development of an uncontrollable need for alcohol, and their inability to abstain. In contrast, alcohol dependence in the remaining subtypes, which collectively are called secondary addicts, develops in the course of prolonged drinking.
- In steady endogenous symptomatic drinkers, alcoholism is secondary to a major psychiatric disorder. Subtypes of this category include schizoid, schizophrenic, and syphilitic alcoholics.
- Intermittent endogenous symptomatic drinkers are distinguished primarily by their periodic drinking pattern but also develop alcoholism secondary to a psychiatric disorder. For example, epileptic and epileptoid drinkers are driven to wild drinking bouts by a seizure-like brain disorder. Similarly, manic-depressive disorder is thought to produce periodic excessive drinking. For so-called hypothetical true dipsomaniacs, periodic drinking is symptomatic of an underlying organic disease.
- In so-called stammtisch drinkers, alcoholism is precipitated by exogenous causes. These people, who can be further subdivided into social compensating, easy-going, and promotional alcoholics, use alcohol on a daily basis around the table (“stammtisch”) set aside for the regular customers at a cafe, bar, or restaurant.

Despite the historical scope and conceptual depth of the Bowman and Jellinek synthesis of typological theory, their classification system inspired virtually no research and received little attention in the subsequent alcohol literature. Nevertheless, two decades later Jellinek (1960*b*) used his familiarity with the world typology literature to make typology theory the centerpiece of his book *The Disease Concept of Alcoholism*. Based on etiologic elements, alcoholic process elements (e.g., level of tolerance or loss of control), and damage elements, Jellinek (1960*a*, *b*) proposed five types, or species, of alcoholism: alpha, beta, gamma, delta, and epsilon (table 1). Jellinek considered only two of the species—gamma and delta alcoholics—to exhibit sufficient evidence of alcohol dependence to represent true disease entities. These two types differ primarily in terms of etiologic factors (i.e., gamma alcoholics drink because of psychological vulnerability, whereas delta alcoholics drink because of social and economic influences) and alcoholic process elements (e.g., gamma alcoholics exhibit loss of control, whereas delta alcoholics exhibit an inability to abstain from alcohol consumption).

Jellinek’s new typology still closely resembled the earlier Bowman-Jellinek synthesis. Compared with the older classification system, the 1960 typology combined the two groups of symptomatic drinkers into one group, the gamma alcoholics; renamed the primary addicts (sometimes called true dipsomaniacs) as epsilon alcoholics; and designated the more severe stammtisch drinkers as delta alcoholics. Although the alpha and beta subtypes were implicit in the 1941 classification, it was not until Jellinek added a biobehavioral concept of dependence to the theory that these alcoholism subtypes, which were not characterized by physical dependence, assumed a prominent place in typological classification.

With the tremendous popularity of Jellinek’s (1960*b*) book on the disease concept, the gamma-delta typology became the most widely accepted system for differentiating among types of alcoholics, perhaps because it was imbedded in a credible and comprehensive theory of alcoholism that represented the cumulative contributions of scores of clinicians and scholars. Drawing from the clinical literature published in France, England, Germany, and the United States, and from the growing body of experimental research conducted in the 1940’s and 1950’s, Jellinek’s theory became a standard fixture in the vocabulary of alcohol studies, less for its originality than for its ability to organize complex clinical phenomena into meaningful categories.

Despite the general acceptance of Jellinek’s theory, however, the typology stimulated little empirical research, nor did it inspire attempts to develop comprehensive diagnostic measurements or to match subtypes to specific therapeutic interventions (Babor and Dolinsky 1988). Nevertheless, Jellinek’s work provided typology research with a new impetus that ushered in the post-Jellinek era of typology development.

## The Post-Jellinek Era

Until the 1960’s, typology theory—including Jellinek’s work—was guided primarily by armchair intuition and clinical observation. With the development of better measurement techniques and research methods, however, empirical research on typologies gained momentum. For example, in the a priori comparative approach, researchers classify two or more groups of alcoholics on the basis of defining, or a priori, criteria (e.g., gender, family history of alcoholism, or coexisting psychopathology) and then compare these groups on hypothetical correlates, such as age of onset, rapidity of symptom development, and severity of dependence. Several studies using this approach indicated that alcoholic subtypes defined by single dimensions could indeed be differentiated in predictable ways on a variety of other dimensions (see Babor and Dolinsky 1988). For example, research using gender as a defining typological criterion showed that compared with men, women underwent a later onset of alcoholism and a more rapid course of symptom development and were more likely to experience depression prior to becoming alcohol dependent (Del Boca 1994). The study also demonstrated, however, that various typological criteria other than gender—such as psychopathology, sex-linked physiological characteristics, and socially defined gender roles—could better explain these differences.

A history of alcoholism in first-degree relatives also has been used frequently as a typological criterion in the post-Jellinek period. Several studies found that alcoholics with positive family histories experienced an earlier onset of dependence symptoms, more social and personal problems connected with their drinking, a rapid course of symptom development, and more severe alcohol dependence than alcoholics with negative family histories (Frances et al. 1980; Penick et al. 1978).

Other studies compared alcoholics with and without coexistent psychopathologies. These analyses found, for example, that alcoholics with antisocial personality disorder (ASPD) began drinking earlier, progressed to problem drinking more rapidly, and experienced more complications from their drinking than alcoholics without ASPD (Hesselbrock et al. 1984).

These examples demonstrate that although various typologies use different defining criteria, they often identify similar subgroups of alcoholics. For example, typologies differentiating between late onset and early onset subtypes (Buydens-Branchey et al. 1989; Parrella and Filstead 1988) closely resemble alcoholic subtypes defined by the presence or absence of familial alcoholism, antisocial behavior, or psychiatric disorders.

### Examples of Typologies Developed in the Post-Jellinek Era

Beginning in the 1970’s, typological theorists began to incorporate greater complexity into their models, not only by postulating subtypes that encompass multiple defining characteristics but also by deriving the typological characteristics from empirical data. Examples of these newer, multidimensional typologies include Morey and Skinner’s (1986) hybrid model, Cloninger’s (1987) neurobiological learning model, Zucker’s (1987) developmental model, and Babor and colleagues’ (1992) vulnerability and severity theory, all of which are summarized below.

Morey and Skinner (1986) administered a battery of psychological tests to 725 subjects seeking treatment for alcohol abuse. Using a complicated statistical technique called cluster analysis, which searches for groups of people with similar characteristics, the researchers identified three types of drinkers: early stage problem drinkers, affiliative drinkers, and schizoid drinkers. The first type, early stage problem drinkers, includes people with alcohol-related health and social problems who have not developed major symptoms of alcohol dependence. The second type, affiliative drinkers, are more socially oriented, tend to drink on a daily basis, and demonstrate moderate alcohol dependence. In contrast, schizoid drinkers are socially isolated, drink in binges, and exhibit the most severe dependence symptoms.

Based on prospective adoption studies, Cloninger (1987) and colleagues (1981) proposed a neurobiological learning model of alcoholism that distinguishes two genetic subtypes, termed type I (“milieu limited”) and type II (“male limited”). Type I alcoholics are thought to experience a later onset of alcohol problems, develop psychological rather than physical dependence, and report feelings of guilt about their alcohol use. In contrast, type II alcoholics manifest alcohol problems at an early age, exhibit spontaneous alcohol-seeking behavior, and are socially disruptive when drinking. Heritable personality characteristics, such as novelty seeking, may account for these different types of alcoholism. The age of onset (early versus late) provides a convenient way to classify patients who resemble type I and type II alcoholics (von Knorring et al. 1985; Buydens-Branchey et al. 1989). (For more information on this typology, see the article by Cloninger and colleagues, pp. 18–23.)

Zucker’s (1987) developmental model, which was derived in part from a longitudinal study of 102 alcoholic men, postulates four types of alcoholism—antisocial, developmentally cumulative, negative affect, and developmentally limited—with the following characteristics:

- Antisocial alcoholism is characterized by the early onset of both alcohol-related problems and antisocial behavior. This alcoholism type is thought to have a genetic basis and a poor prognosis.
- In developmentally cumulative alcoholism, drinking initially is limited and induced by cultural influences. Over the life course, however, the cumulative alcohol consumption is sufficient to produce alcohol dependence.
- Negative-affect alcoholism, which is considered to occur primarily in women, is characterized by the use of alcohol for mood regulation and to enhance social relationships.
- Developmentally limited alcoholism is characterized by frequent heavy drinking in late adolescence that tends to remit to social drinking after the individual successfully assumes adult responsibilities, such as a career and a family.

Babor and colleagues (1992) based their typology on the assumption that the heterogeneity among alcoholics is attributable to a complex interaction among genetic, biological, psychological, and sociocultural factors. Consequently, no single characteristic distinguishes alcoholics from non-alcoholics, and separate homogeneous subtypes differ by more than just one defining characteristic. The researchers therefore reviewed the alcoholism typology literature since the mid-19th century to identify defining typological characteristics that combined could accurately describe alcoholic subtypes. Using cluster analysis, the investigators identified two types of alcoholics who differ consistently across 17 defining characteristics, including age of onset, severity of dependence, and family history of alcoholism. One group, designated type A alcoholics, is characterized by later onset of alcoholism, fewer childhood risk factors (e.g., conduct disorder and attention deficit disorder), less severe alcohol dependence, fewer alcohol-related problems (e.g., arrests or job loss), and less psychopathology. The other group, termed type B alcoholics, is characterized by childhood risk factors, a family history of alcoholism, early onset of alcohol-related problems, greater severity of dependence, multiple drug use, a more chronic treatment history despite their younger age, greater psychopathology, and more life stress. The two types also differ with respect to treatment outcome, with type B alcoholics more likely to relapse to heavy drinking.

In general, typology research during the post-Jellinek era has been characterized by the systematic study of clinical populations using a variety of empirical techniques, including psychological testing, clinical interviews, and analysis of biological markers. Combining these techniques with innovative research designs—such as genetic epidemiology; prospective, longitudinal monitoring; and post-treatment followup evaluations—modern typology research has led to an improved conceptual understanding of the complex array of variables characterizing the diversity among alcoholics. Moreover, as typologies based on single defining characteristics (e.g., gender or family history of alcoholism) have given way to multidimensional classification schemes, researchers for the first time have conducted replication studies.

Despite these significant improvements in recent typology research, the field still faces some challenging issues. For example, perhaps because of the differences in measurement techniques and methodological approaches, typology researchers have not always recognized the similarities between their own work and that of other investigators. And although some theories are likely to endure longer than others, a more fundamental question remains concerning the utility of typologies for theory development and clinical practice.

## The Past as Prologue: Whither Typology Theory?

As this review has outlined, throughout the past 150 years, researchers and clinicians have developed numerous typological classifications of alcoholism. These classifications have distinguished alcoholism subtypes based on a multitude of defining characteristics, including drinking patterns, consequences of drinking, personality characteristics, and coexisting psychiatric disorders. Despite the variety of determining factors and manifestations of alcoholism and despite the inconsistencies in nomenclature, however, both clinical observation and empirical research indicate that the heterogeneity among alcoholics is not random. As shown in table 2, similar alcoholic subtypes can be categorized within two broad groups, called the Apollonian and Dionysian types, based on recurrent characteristics of the drinkers. This means that, for example, type A alcoholics are basically the same as milieu-limited or delta alcoholics, with some differences between these types resulting from the different methods and defining criteria used to establish the typologies.

The Apollonian-Dionysian distinction has been used to summarize the commonalities among alcoholic subtypes. Greek and Roman mythology attributes the characteristics of contemplation, intellect, artistic creativity, and self-restraint to the god Apollo. As suggested in the subtypes grouped under this designation, when alcohol dependence develops in such an individual, typically after years of socially approved heavy drinking, it presents in a more benign form. Consequently, Apollonian subtypes include alcoholics who are characterized by later onset, a slower disease course, fewer complications, less psychological impairment, and a better prognosis. In contrast, the god Dionysius was known for his drunken revelry, sexual abandonment, and physical aggression. When alcohol dependence develops in this type of personality, it can be identified by the subtype characteristics of pathological drinking and drunken comportment. Thus, Dionysian subtypes of alcoholics are characterized by early onset, more severe symptomatology, greater psychological vulnerability, and more personality disturbance.

It is interesting to note that the subtypes summarized in table 2 have been identified through armchair intuition as well as by comparative research and empirical clustering techniques. To the extent that different methods have identified subtypes with similar features, this provides strong evidence for the cumulative wisdom of the past as well as the progress made in recent years.

Confirmation of the hypothesis that only two broad categories of alcoholics exist would represent an important breakthrough for theory development and treatment matching. For example, research on the etiology of alcoholism might be informed by the possibility that two different paths may lead to alcohol dependence—one originating primarily in environmental influences and the other in genetic and personality factors. Treatment matching and patient placement also might profit from this knowledge, provided that different therapeutic approaches and treatment settings prove to be differentially effective with different types of alcoholics. Despite one-and-a-half centuries of progress and a remarkable acceleration of interest in alcohol research in the past two decades, these critical issues continue to define the challenge as well as the promise of typology theory.

## Figures and Tables

**Table 1:** Characteristics of Four of Jellinek’s Species of Alcoholism^1^,^2^

Characteristics	Species			

	Alpha	Beta	Gamma	Delta
**Etiological Elements**				
Psychological vulnerability	High	Low	High	Low
Physiological vulnerability	Low	Low	High	High
Sociocultural influences	Low to moderate	Low to moderate	Low to moderate	High
Economic influences	Low to moderate	Low to moderate	Low to moderate	High

**Alcoholic Process Elements**				
Nature of dependence	Psychological	No dependence	Psychological, then physical	Physical, then psychological
Acquired tissue tolerance	Low	Low	High	High
Loss of control	Low^3^	Low	High	Low
Inability to abstain	Low	Low	Low	High
Progression	Slight	Slight	Marked	Slow
Nutritional/physical habits	Good to poor	Poor	Poor	Fair

**Damage Elements**				
Physical/mental	Low to moderate	High	Low to high	Low to high
Socioeconomic	Low to moderate	Low	High	High

1 Adapted from Jellinek 1960*a*.

2 Epsilon alcoholism, the fifth species, is not included in this table because Jellinek considered knowledge of that subtype to be too scant to describe in detail.

3 According to Jellinek, alpha alcoholism is characterized by deliberate undisciplined drinking.

SOURCE: Babor and Dolinsky 1988.

**Table 2:** Chronological Compendium of Typological Theories Organized According to Apollonian and Dionysian Characteristics

Theorist	Year	Apollonian Types	Dionysian Types
Carpenter	1850	Chronic	Periodic
LeGrain	1889	Weak willed	Morally insane
Kerr	1893	Habitual	Periodic
Triboulet and Mathieu	1900	Habitual drinkers	Regular alcoholics and dipsomaniacs
Crothers	1911	Continuous Acquired	Periodic and dipsomaniacs Hereditary
Wingfield	1919	Chronic sober	Chronic inebriates and dipsomaniacs
Kretschmer	1924	Cyclothymic	Schizoid
Knight	1938	Reactive	Essential, symptomatic
Bowman and Jellinek	1941	Stammtisch	Steady symptomatic, periodic symptomatic, and primary alcoholics
Jellinek	1960	Delta	Gamma
Frances et al.	1980	Family history negative	Family history positive
Morey and Skinner	1986	Affiliative drinkers	Schizoid drinkers
Cloninger	1987	Milieu limited	Male limited
Zucker	1987	Developmentally cumulative	Antisocial and negative affect
Buydens-Branchey et al.	1989	Late onset	Early onset
Babor et al.	1992	Type A	Type B

## Footnotes

1 For a definition of this and other technical terms used in this article, see the glossary, p. 8.

2 Treatment matching refers to the concept that alcoholics with specific characteristics will benefit most from certain treatment approaches.

## Glossary

Alienist: A physician who specializes in the treatment of mental disorders and in the management of mental institutions.
Apomorphine: A substance derived from morphine and codeine that sometimes was administered as a nausea-inducing agent in aversion therapy for alcoholism and to relieve alcohol craving temporarily.
Atropine: An extremely poisonous, bitter organic compound sometimes used medically with *strychnine* to reduce alcohol craving.
Cyclothymia: A personality pattern marked by alternating periods of elation and sadness, activity and inactivity, and excitement and depression.
Dipsomania: An irritability of the nervous system characterized by alcohol craving. In pseudodipsomania, an initial drink of alcohol produces immediate craving and tremendous bouts of excessive drinking. In true dipsomania, craving occurs spontaneously and does not require alcohol to excite it.
Dyspepsia: Disturbed digestion; indigestion.
Inebriety: A constitutional disease characterized by a very strong morbid impulse to drink or crave for alcohol.
Melancholia: A pathological state in which the individual is depressed, unresponsive to most stimuli, and seems sad without apparent cause.
Moral insanity: A condition in which all sense of right and wrong as well as duty and obligation is feeble or wanting.
Morphia: The principal compound of opium, used medically as a narcotic, pain-relieving agent during alcohol withdrawal.
Narcomania: An inexpressibly intense, involuntary morbid craving for the temporary anesthetic relief promised by all forms of narcotics.
Neurasthenia: Exhaustion of the nervous system, considered a cause of certain forms of *inebriety.*
Oinomania: Wine mania; an inordinate and uncontrollable thirst for excessive quantities of intoxicating drinks.
Psychasthenia: A form of neurosis marked by morbid anxiety, fixed ideas, and obsessions.
Strychnine: An extremely poisonous organic compound used medically as a stimulant and as a cure for alcohol craving.
